# Mucoadhesive chitosan-coated boswellic acids nanoparticles as promising gastroprotective nanoagents via modulation of the RAS/ERK signaling pathway

**DOI:** 10.1186/s11671-025-04375-8

**Published:** 2025-10-23

**Authors:** Reem T. Attia, Asaad Babker, Mohamed S. Nafie, Fatma Aboelmagd Saleh, Manar Ashraf Abdelwareth, Esraa Mahmoud Loutfy, Ayman Ali Mohammed Alameen, Sherif Ashraf Fahmy

**Affiliations:** 1 Department of Pharmacology, Toxicology, and Biochemistry, Faculty of Pharmacy, Future University in Egypt (FUE), Cairo, Egypt; 2 Department of Medical Laboratory Sciences, College of Health Sciences, Gulf Medical University, Ajman, United Arab Emirates; 3 Department of Chemistry, College of Sciences, University of Sharjah, P.O. 27272, Sharjah, United Arab Emirates; 4 Chemistry Department, Faculty of Science, Suez Canal University, P.O. 41522, Ismailia, Egypt; 5 Faculty of Pharmacy, Future University in Egypt (FUE), Cairo, Egypt; 6 Department of Clinical Laboratory Sciences, College of Applied Medical Sciences, Jouf University, Sakaka, Saudi Arabia; 7 Department of Chemical Pathology, Faculty of Medical Laboratory Sciences, University of Khartoum, Khartoum, Sudan; 8 Department of Pharmacy, Institute of Pharmaceutics and Biopharmaceutics, Marburg University, Robert-Koch-Str. 4, 35037 Marburg, Germany

**Keywords:** Boswellic acids, Chitosan, Nanoparticles, Ulcer

## Abstract

Chitosan, a natural biological macromolecule, was employed to coat boswellic acids extract nanoparticles (CT/BA NPs) to enhance their gastroprotective potential. This study evaluated the therapeutic effects of CT/BA NPs compared to free boswellic acids (BA) in a murine model of ethanol-induced gastric ulcers. Histological analysis showed that CT/BA NPs preserved gastric gland integrity and minimized inflammatory infiltration, achieving a ~ 97% reduction in ulcer index compared to the ulcer group, whereas BA alone achieved ~ 80% protection. CT/BA NPs treatment markedly modulated key growth factors, with TGF-α and TGF-β1 increased by more than 100% relative to the ulcer group, while VEGF expression was suppressed by ~ 74%, indicating more controlled angiogenesis. In addition, CT/BA NPs significantly reduced RAS and ERK levels (by ~ 1.6- and 1.2-fold, respectively) and lowered TNF-α compared to BA, highlighting their role in regulating the RAS–ERK signaling pathway. The superior mucoadhesive properties of CT/BA NPs (76.4% vs. 15.8% for BA NPs) improved gastric mucosal retention, contributing to localized therapeutic effects. Collectively, these findings demonstrate that CT/BA NPs provide promising gastroprotection by promoting epithelial healing, modulating growth factors and signaling pathways, and mitigating ulcer severity.

**Supplementary Information:**

The online version contains supplementary material available at 10.1186/s11671-025-04375-8.

## Introduction

Peptic ulcer disease is a common gastrointestinal disorder characterized by mucosal erosion in the stomach lining, often resulting from an imbalance between aggressive factors such as gastric acid and pepsin and protective mechanisms, including mucus secretion and bicarbonate production[1]. The primary causes of gastric ulcers include Helicobacter pylori (*H. pylori*) infection, prolonged use of nonsteroidal anti-inflammatory drugs (NSAIDs), excessive alcohol consumption, and stress [2]. Gastric ulcers are a global health concern, affecting millions of people annually, with a prevalence of approximately 5–10% worldwide[3]. While incidence rates have declined in developed nations due to improved hygiene and medical treatments, developing countries continue to report high prevalence rates due to persistent H. pylori infections and widespread NSAID use [4]

Pharmacological interventions primarily involve proton pump inhibitors (PPIs), histamine-2 receptor antagonists (H2RAs), and antacids, which reduce gastric acid secretion and promote mucosal healing [5]. Additionally, cytoprotective agents like sucralfate and prostaglandin analogs, such as misoprostol, enhance gastric mucosal defense mechanisms [6]. However, the long-term use of these drugs poses potential side effects, necessitating the exploration of natural compounds with gastroprotective properties [7]. Alternative, safer, and more effective approaches are urgently needed to overcome drug-drug interactions and adverse effects.

Among various natural biopolymers, chitosan, a natural cationic polysaccharide, has attracted considerable attention due to its mucoadhesive properties, biocompatibility, and ability to promote gastric mucosal healing. In addition, several natural therapeutics have garnered significant interest because they stabilize epithelial cells, prevent unwarranted gastric secretions, or augment mucosal shield mechanisms [7]. Boswellia acids (BA), derived from the oleogum resin of the *Boswellia sacra* trees, are a bioactive compound known for its potent anti-inflammatory and antioxidant properties [8]. The therapeutic potential of Boswellia acid stems from its ability to inhibit inflammatory mediators such as leukotrienes and cyclooxygenase enzymes, making it a promising candidate for gastroprotection [9]. Several studies have investigated the gastroprotective properties of *Boswellia* species, attributing their ulcer-preventive effects to their high content of bioactive Boswellic acids (BA) [10]. These compounds exert anti-inflammatory effects by modulating key cytokines, particularly tumor necrosis factor-alpha (TNF-α), which plays a central role in ulcer pathogenesis. TNF-α promotes mucosal inflammation, apoptosis, and gastric acid hypersecretion, contributing to ulcer progression [2]. Several growth factors contribute to gastric mucosal repair, notably transforming growth factor-alpha (TGF-α), transforming growth factor-beta (TGF-β), and epidermal growth factor (EGF). TGF-α and TGF-β stimulate epithelial cell proliferation and exert anti-inflammatory effects, while EGF promotes angiogenesis and tissue regeneration, collectively accelerating ulcer healing [11, 12] Additionally, the rat sarcoma viral oncogene homolog (RAS)-extracellular signal-regulated kinase (ERK) pathway plays a vital role in cell survival and mucosal defense, while vascular endothelial growth factor (VEGF) enhances angiogenesis, ensuring adequate oxygen and nutrient supply to ulcerated tissues [13]. Given the potential of natural compounds to modulate critical healing pathways, strategies to enhance their bioavailability and mucosal retention are highly sought after. Nanotechnology-based approaches, particularly nanoprecipitation, offer an effective means to overcome these limitations. The nanosizing of various therapeutic agents was reported to increase their therapeutic activity while enhancing the mucosal membrane’s penetration [14, 15]. Nanoprecipitation is a facile and efficient approach to engineering various nanoparticles via solvent-shifting [16], enabling the creation of complex polymeric colloidal morphologies. Chitosan (CT) is a natural cationic polymer with several attributes that promote its use as a promising coating material for nanoparticles. Coating nanoparticles with CT offers multiple advantages, including biocompatibility, biodegradability, mucoadhesiveness, enhanced mucus membrane penetration, and improved stability [17].

To harness these mechanisms, we developed a novel chitosan-coated Boswellic acids nanoparticulate system (CT/BA NPs). Then, we systematically evaluated its size, surface charge, morphology, yield percentage, stability, chitosan conjugation efficiency, and mucoadhesive capability. Furthermore, we explored the potential of using Boswellic acids and their chitosan-coated nanoparticles to prevent ethanol-induced gastric ulcers in vivo.

## Materials and methods

### Materials

Oleo gum resins were brought from *Boswellia sacra* plants cultivated in Oman. Low molecular weight chitosan (CT) was acquired from Biosynth Carbosynth (Berkshire, UK). All other chemicals were bought from Sigma-Aldrich (St. Louis, MO, USA).

### Methods

#### Extraction and characterization of the major phytochemicals of *Boswellia sacra* oleogum resin

Methanol was utilized to extract the Boswellic acids of the gum resin, forming methanolic extract as described in our previous study. The extracted compounds were analyzed employing liquid chromatography-electrospray ionization-tandem mass spectrometry (LC/ESI–MS/MS) with an X500R LC-QTOF mass spectrometer (SCIEX, USA) involving Inertsil C18 column (25 cm × 4.6 mm × 5 µm), as described in our reported study [18, 19]

#### Preparation of BA NPs and CT/BA NPs

Boswellic acids extract nanoparticles (BA NPs) were engineered using the nanoprecipitation approach, as shown in previous reports with little adaptations [20, 21], where the solvent-shifting strategy disperses hydrophobic compounds in the aqueous phase. In brief, the organic phase, formed by dissolving 200 mg of frankincense extract in ethanol, was added to the aqueous phase, consisting of 0.5% polyvinyl alcohol (0.5%) in distilled water. The organic phase was added to the aqueous one dropwise while magnetic stirring was conducted. The formed colloidal dispersion was ultrasonicated for 15 min, followed by magnetic stirring for 12 h at room temperature to eliminate the remaining ethanol. Then, chitosan-coated BA NPs (CT/BA NPs) were produced to impart the bioadhesive characteristics by dropwise addition of CT solution (0.25%) prepared in 1% *v*/*v* glacial acetic acid to the BA NPs dispersion while steadily stirring. Afterwards, the final solution was sonicated for 10 min in a bath sonicator and then magnetically stirred for 2 h at room temperature. All solutions were kept at 4 °C for further evaluation [22]

#### Characterization of the NPs

The average particle size, size distribution, and zeta potential of the freshly prepared NPs were analyzed employing a Malvern Zetasizer (Malvern Instruments, Worcestershire, UK) at 25 °C [22, 23]. The surface morphology of the NPs was explored using transmission electron microscopy (JEOL JEM-2100, Musashino, Akishima, Tokyo, Japan) working at 140 kV. The samples were set as described previously [24, 25].

#### Yield percentage (Y%) of BA NPs

The Yield % of BA NPs was determined by centrifuging the colloids at 18,000 rpm for 1 h, followed by discarding the supernatant[26]. Then, the pellets were dried and weighed. The Yield percent of each formula was calculated using Eq. 1.

1 $$ Yield\;\;Percentage\;\left( \% \right) = \;\frac{Actual\;\;Yield\;}{{Theoretical\;\;Yield\;}}\; \times \;100$% MathType!End!2!1! $$

#### Percentage of conjugated (Conj%) CT

The CT % conjugated to BA NPs was assessed using the indirect method [27]. Briefly, a 2 ml sample was centrifuged at 15,000 rpm for 1 h at 4 °C. Afterward, the UV absorbance of the supernatant was measured at 215 nm using a Shimadzu UV-1800 spectrophotometer (Japan). Equation 2 was then used to calculate the conjugation %.

2 $$ Conjugation\;\;Percentage\;\left( \% \right) = \;\frac{Total\;\;CT\; - \;Unconjugated\;\;CT\;}{{Total\;CT}}\; \times 100$% MathType!End!2!1! $$

#### Stability study

The stability of the BA NPs and CT/BA NPs was assessed by storing them in the fridge at room temperature (4 °C and 25 °C) for 4 months. Then, at defined time intervals (1, 2, 3, 4, and 6 weeks), the average sizes, PDI, and surface charges were determined [25, 28].

#### Mucoadhesive efficiency (ME%)

The ME% was assessed utilizing the adsorption method, as detailed previously, with some modifications [22, 29]. The free BA, BA NPs, and CT/BA NPs were mixed with an equal volume of mucin solution prepared in phosphate buffer saline of pH 7.4 in a 1 mg/mL concentration. The mixtures were then subjected to magnetic stirring and incubated at 37 °C inside an oven for 120 min. Afterwards, each mixture solution was centrifuged at 10,000 rpm for 120 min, and then the supernatant was collected and filtered. Then, the concentration of free mucin was estimated spectrophotometrically (at λ_max_ 260 nm), and the concentration of mucin adsorbed was calculated in proportion to Eq. 3.

3 $$ {\text{ME\% }} = \frac{{{\text{Initial\;}}\;{\text{concentration\;}}\;{\text{of}}\;{\text{\;mucin\;}} - {\text{\;the\;}}\;{\text{concentration\;}}\;{\text{of}}\;{\text{\;free\;}}\;{\text{mucin}}}}{{{\text{Initial}}\;{\text{\;concentration\;}}\;{\text{of\;}}\;{\text{mucin\;}}}} \times 100$% MathType!End!2!1! $$

### Animals and experimental design

Twenty-four male Swiss albino mice (20–30 g) were purchased from the National Research Center (Cairo, Egypt). The study protocol was approved by the Ethical Committee for Animal Experimentation at Future University in Egypt (FUE), permit number “REC-FPFUE-17/2023”. Animals were housed under normal laboratory conditions (22 ± 2 °C; 12 h/12 h light/dark cycle and free access to food and water, the mice were fasted for 24 h with free access to water and were distributed into 4 different groups (n = 6/group) as follows: Control (C): mice only administered distilled water (5 ml/Kg), Gastric Ulcer untreated group (GU): mice administered distilled water by oral gavage (5 ml/Kg) 1 h before disease induction of acute ulcer by 95% ethanol. BA group **(**250 mg/Kg): mice were treated by oral gavage with BA at 250 mg/kg 1 h before 95% ethanol-induced acute ulcer, CT/BA NPs group: mice were treated by oral gavage CT/BA NPs at (250 mg/kg) 1 h before the induction of acute ulcer using 95% ethanol. The mice were sacrificed 4 h after treatment with absolute ethanol [30]. The sample size (n = 6/group) was selected based on ethical compliance with the 3Rs principle (Replacement, Reduction, Refinement), minimizing animal use while ensuring statistical validity. This was also selected in alignment with similar validated experimental models in the literature [2, 10, 20].

### Biochemical analysis

The animals were sacrificed using a lethal dose of thiopental (IP 200 mg/kg). Then, the gastric mucosae were dissected out on an ice/salt mixture, weighed, and homogenized in phosphate-buffered saline to prepare 10% homogenate. The clear supernatants were separated into aliquots to determine the targeted pathway proteins with the corresponding ELISA kits according to the manufacturer’s instruction: Tumor necrosis factor-α (TNF-α; Catalogue #: MBS355371), Extracellular Signal-Regulated Kinase (*P*-ERK, Catalogue #: MBS2511166), Epidermal growth factor(EGF; Catalogue #: MBS824918), transforming growth factor-alpha (TGF-α; Catalogue #: MBS765046), transforming growth factor-beta (TGF-β1; Catalogue #: CSB-E04727r), Rat Sarcoma (RAS; Catalogue #: MBS038125) (*MyBioSource Inc., CA, USA; CUSABIO, TX, USA).*

### Determination of ulcer and preventive indices

Following euthanasia, the stomachs of all animals were carefully dissected, opened along the greater curvature, and rinsed with cold saline to remove gastric contents. The inner surface of each stomach was then examined for visible lesions and photographed against a calibrated millimeter grid background. Briefly, the gastric mucosa was examined for erosion sites, then the ulcer score was assessed according to this scale; a scale of 0–5, where 0 means no injury, 1 means minor hemorrhagic erosion, 2 means gastric ulcer less than 1 mm, 3 means gastric ulcer of 1–2 mm, 4 means gastric ulcer of 3–4 mm and 5 means gastric ulcer more than 4 mm. [31–33]. To quantify the extent of mucosal damage, the ulcerated area (mm^2^) and total gastric mucosal area (mm^2^) were determined by manually counting the number of 1 mm^2^ grid squares. The ulcer index (UI) for each animal was calculated as mentioned previously [2, 34] using the following equation:

$$ Ulcer\;\;index = \frac{{Ulcerated\;\;area\;\left( {m{m^2}} \right)}}{{Total\;gastric\;\;mucosal\;\;area\;\left( {m{m^2}} \right)}}$% MathType!End!2!1! $$

This method allowed for objective quantification of mucosal injury severity. Ulcer index (UI) values were then used to compare the protective effects of the tested treatments across experimental groups. Subsequently, the net preventive index was calculated according to the following equation [35]:

$$ Preventive\;index = \frac{{100 \times \;\left( {UI\;\;of\;\;GU\;\;group - UI\;\;of\;\;treated\;\;group} \right)}}{UI\;\;of\;\;GU\;\;group}$% MathType!End!2!1! $$

## Histological and immunohistochemistry analysis

The gastric mucosa was fixed in 10% buffered formalin for 24 h. Dehydration was accomplished by using methyl, ethyl, and 100% ethyl alcohol dilutions in sequence, following washing. The specimens were immersed in paraffin and then rinsed with xylene before being heated to 56 °C in a hot air oven for 24 h. Tissue blocks made of paraffin and beeswax were sectioned at a thickness of 4 microns using a sledge microtome. The obtained tissue sections were collected on glass slides, deparaffinized, and stained with hematoxylin and eosin stain (H&E) for examination using the light microscope. For immunohistochemical detection, formalin-fixed, paraffin-embedded (FFPE) sections of mouse gastric mucosa (4 μm) were deparaffinized, rehydrated, and subjected to heat-induced antigen retrieval using sodium citrate buffer (pH 6.0) at 95–98°C for 15–20 min. Endogenous peroxidase was blocked with 3% H₂O₂, followed by blocking with 5% normal goat serum. Sections were incubated overnight at 4°C with a mouse monoclonal anti-VEGFA antibody (MyBioSource, MBS438814, 1:200), followed by a 30-min incubation with an HRP-conjugated goat anti-mouse secondary antibody (MyBioSource, MBS198064, 1:200). VEGF was visualized using 3,3′-diaminobenzidine (DAB) chromogen, and nuclei were counterstained with Mayer’s hematoxylin. Slides were dehydrated, cleared, and mounted for microscopic evaluation. Immunoreactivity was assessed using ImageJ software, with color deconvolution applied to quantify VEGF expression. Positive controls (VEGF-rich tissues) and negative controls (isotype or antibody omission) confirmed staining specificity [10, 36].

### Toxicity assessment: hematological and biochemical analysis of blood samples

Blood samples were collected from the control, ethanol-induced gastric ulcer mice (GU), BA, and CT/BA NPs treated mice (n = 6). The complete blood count (CBC) was evaluated, including red blood cells, white blood cells, and hemoglobin counts, employing Abbott CELL-DYN^®^ 1800 automated hematology analyzer (USA) and commercially available kits (Abbott Laboratories, USA) [37]. In addition, the levels of ALT and AST were determined in the serum samples utilizing commercial assay kits.

### Statistical analysis

The data were displayed in the form of the mean ± the standard deviation. Analysis of Variance (One-way ANOVA) test, followed by Tukey multiple comparison tests, was performed to assess statistical significance using GraphPad Prism software version 10.4.1 (GraphPad Software, San Diego, CA). In all experiments, the statistical level of significance was set at *p* value ≤ 0.05.

## Results and discussion

### LC/ESI–MS–MS analysis of the Boswellia extract

The major chemical compounds of Boswellia extract were explored employing LC–MS/MS in negative high-resolution ESI mode. The descriptive base peak chromatograms were previously reported [18] and are displayed in Figure S1 and Table S1. Data handling for the major ingredients relied on precise molecular mass and MS/MS fragment ions, contrasted with data from the NIST Library. It has been revealed that three significant triterpenic acids were found in the extract, namely Boswellic acids (BA), which are 11-keto-beta-boswellic acid, maslinic acid, and 3-acetyl-11-keto-beta-boswellic acid. Several studies have reported promising anti-inflammatory, anticancer, and inflammatory bowel disease effects of BA, in addition to its preventive and healing effects of boswellic acid in gastric ulcers. This makes them potential prophylactic and therapeutic agents in the case of gastric ulcers, especially for alcohol/nonsteroidal anti-inflammatory drugs (NSAIDs)-induced ulcers [38–40]. The anti-ulcerative activity of BA is attributed to its leukotriene (LTB4) inhibiting capability[41]. In order to further improve the anti-ulcerative activity of BA, they were nanosized employing the solvent-shifting technique and coated with mucoadhesive CT film to enhance their gastric mucoadhesion power.

### Characterization of the NPs

**Fig. 1:**
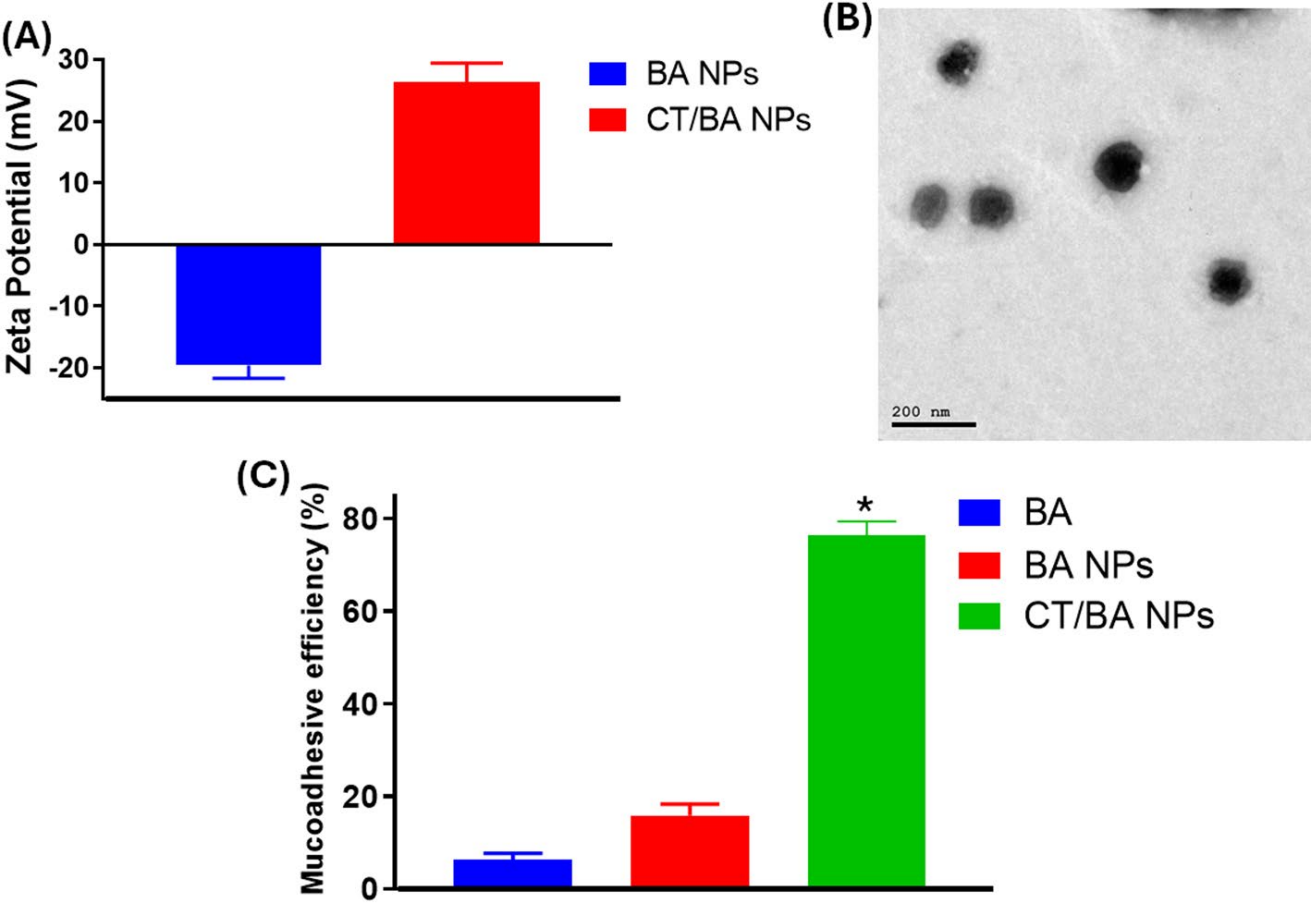
**A** Zeta potential of BA NPs and CT/BA NPs, **B** TEM image of CT/BA NPs, and **C** Mucoadhesive efficiency (%) of BA, BA NPs, and CT/BA NPs. *Significant results (*p* < 0.05) compared to BA and BA NPs

The physicochemical evaluation of the designed NPs emphasized the influence of CT decoration on nanoparticles' properties (Table 1 and Fig. 1A). The BA NPs had an average particle size of 119.80 ± 6.30 nm, while the CT/BA NPs exhibited a bigger size of 132.41 ± 5.70 nm. This increment in average size evidences the successful coating of the NPs with CT [42]. Moreover, the BA NPs and CT/BA NPs had PDI values of 0.12 ± 0.05 and 0.15 ± 0.03, respectively. These findings showed a relatively narrow size distribution, implying the outstanding homogeneity of the NPs, even after being coated with CT. The morphological analysis is important in defining the ability of the NPs to adhere to the cell surface. Our findings showed that the designed CT/BA NPs had a spherical structure with minimal aggregations. The small particle size of the formulated CT/BA NPs could allow efficient biodistribution and cellular uptake [33].

**Table 1:** The average size, polydispersity index (PDI), Yield percentage (Y%), and conjugated chitosan percentage (Conj. %) of the designed NPs. Data are given as mean ± SD; n = 3

Samples	Average Size (nm)	PDI	Y (%)	Conj. (%)
BA NPs	119.80 ± 6.30	0.12 ± 0.05	93.8 ± 4.1	–
CT/BA NPs	132.41 ± 5.70	0.15 ± 0.03	–	95.1 ± 6.7

**Fig. 2:**
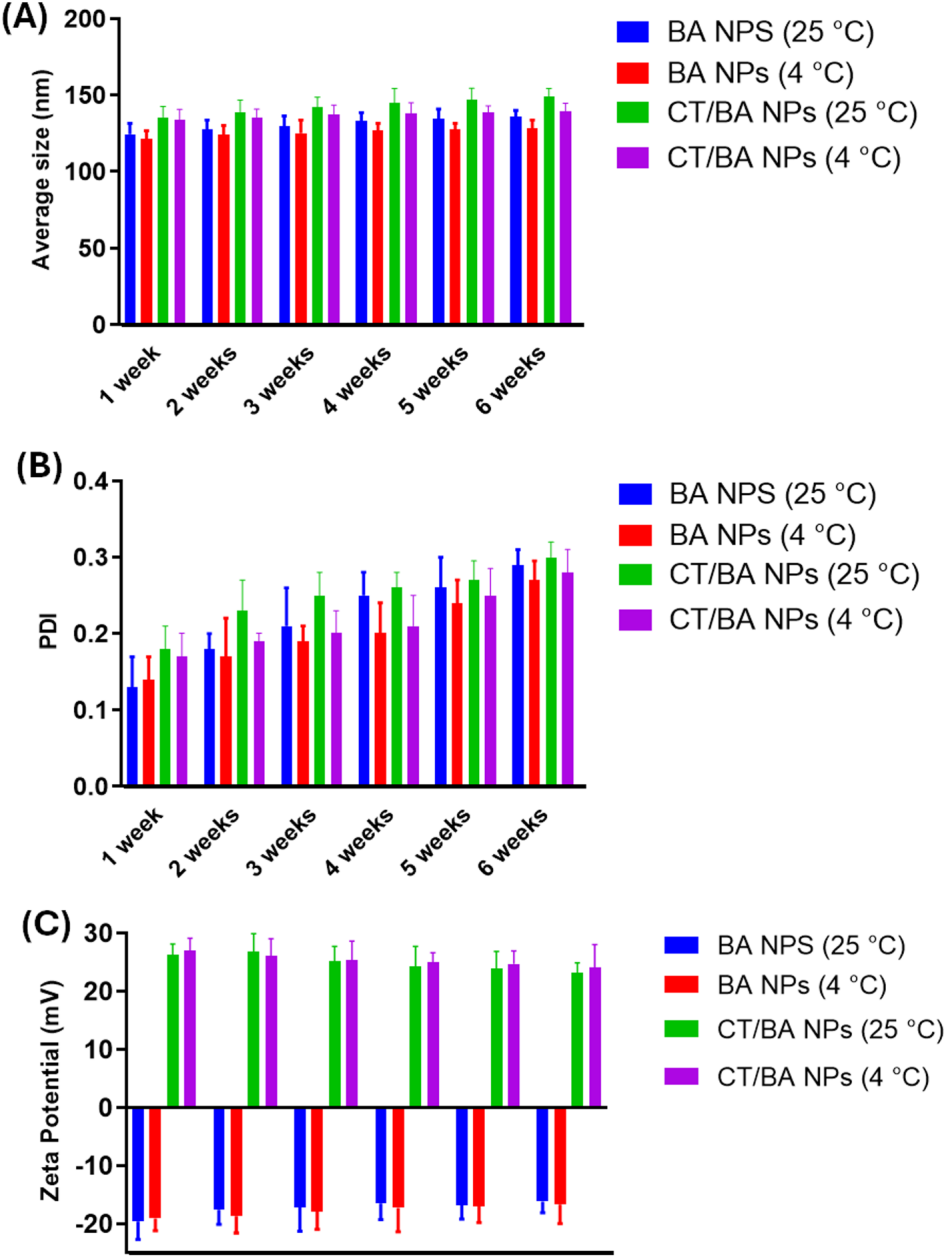
Storage stability over 6 weeks at 25 °C and 4 °C is presented as **A** average size, **B** PDI, and **C** zeta potential

Distinctive differences between the surface charges of BA NPs and CT/BA NPs were observed upon zeta potential examination (Fig. 2A). The uncoated NPs had a zeta potential of -19.6 ± 2.1 mV that was, inversely, increased to 26.3 ± 3.2 mV after decoration with CT. This increment in the surface charge is credited to the coating with the cationic polymer, CT, that constitutes a cationic ammonium group within its structure. This evidence shows the effective coating of the BA NPs with CT via electrostatic interactions [42]. Thus, coating BA NPs with CT imparts positive charges to them, facilitating their conversion into cationic NPs that could interact with anionic gastric cell membranes as well as improve their stability. [29].

The engineered NPs were kept for up to 6 weeks at various temperatures (25 °C and 4 °C), and physicochemical characteristics were evaluated at various time intervals (1, 2, 3, 4, and 6 weeks). Our findings suggested the stability of our preparations over the experiment period since only minimal changes were observed regarding the average size, PDI, and zeta potential (Fig. 2A–C) Moreover, the Y% of the BA NPs was found to be 93.8 ± 4.1, implying that the solvent-shift method effectively produced the BA NPS. Whereas the CT Conj. % of CT/BA NPs was 95.1 ± 6.7, suggesting the successful coating of the NPs with CT film. The high Conj.% of CT is critical for ensuring the mucoadhesive power and cationic nature of the NPs, thereby potentially improving the adhesion of the NPs to the gastric cells and exposing the constricted junctions of epithelial cells and thus advancing the paracellular penetrability [22, 29].

### Mucoadhesive efficiency (ME%)

The mucoadhesive efficiencies of BA, BA NPs, and CT/BA NPs were examined relying on the mucin adsorption attributes, and the findings are depicted in Fig. 1C. Our findings showed that the ME% of CT/BA NPs (76.4 ± 2.9%) was significantly higher than that of BA (6.2 ± 1.5%) and BA NPs (15.8 ± 2.5%). This is attributed to the electrostatic interaction between the cationic CT and anionic mucin, increasing the binding efficiency of the coated BA NPs to mucin [43]. These findings collectively suggest that coating the BA NPs with CT film has increased their mucoadhesive features and their residence time in the gastrointestinal tract, enhancing the anti-ulcerative activity [44].

### Ulcer and preventive indices

**Fig. 3:**
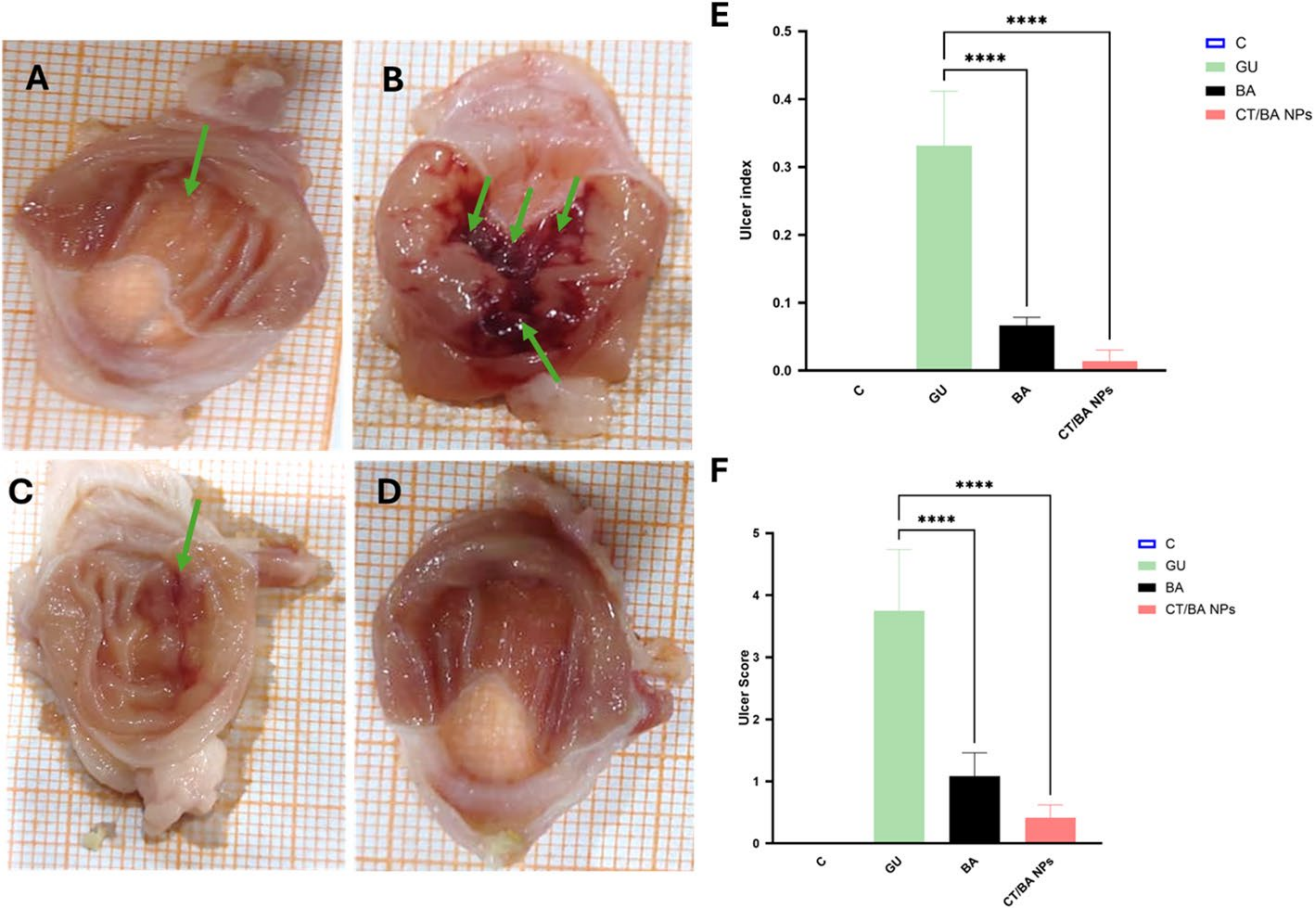
Macroscopic images of gastric mucosa in mice: **A** Control group showing normal mucosa with no lesions, **B** Gastric ulcer GU group displaying extensive hemorrhagic and necrotic lesions, **C** BA-treated group showing mild mucosal streaking and localized irritation, **D** CT/BA NPs treated group showing preserved mucosal integrity with no visible ulcers, **E** Ulcer index and **F** Ulcer score values. The results are shown as mean n = 6 ± SD (*) shows a significant difference from BA group

In our formulation, the nanoparticles themselves serve as therapeutic agents without any additional encapsulated or adsorbed drugs. This approach aligns with the emerging field of self-therapeutic nanomaterials, where the intrinsic properties of the nanoparticles confer therapeutic effects. Similar methodologies have been reported in recent literature, where the therapeutic efficacy of such nanoparticles is attributed to their inherent characteristics rather than the release of a separate drug component. The current study evaluated the gastroprotective effects of BA and its nanoformulation (CT/BA NPs) on experimentally induced gastric ulcers. The assessment was performed using macroscopic ulcer scoring, ulcer index, and preventive indices calculations. Macroscopic examination of gastric tissues revealed notable differences in mucosal integrity across experimental groups. The control group (Fig. 3A) exhibited intact gastric mucosa with no visible lesions, confirming normal physiological conditions. In contrast, the gastric ulcer (GU) group (Fig. 3B) demonstrated extensive hemorrhagic lesions covering a substantial portion of the mucosa, resulting in a significantly elevated ulcer index indicative of severe ulceration. Treatment with the bioactive agent (BA) alone (Fig. 3C) moderately reduced mucosal damage, showing only minor streaks and focal redness along with visible congestion, corresponding to a significantly reduced ulcer index. The CT/BA NPs showed promising protection of the gastric mucosa, with an almost normalized ulcer index close to the control group (Fig. 3D). These findings suggest that CT/BA NPs offer superior gastroprotection compared to BA alone. As expected, the GU (gastric ulcer) group exhibited the most severe mucosal damage, as evidenced by a significantly elevated ulcer score of (3.75 ± 0.98) and ulcer index (0.33 ± 0.08). These findings are consistent with previously reported models of ulcer induction, where tissue injury is characterized by deep hemorrhagic lesions, surface erosions, and necrosis. The presence of deep ulcers and hemorrhagic streaks in this group confirms the validity of the model used [45]. The CT/BA NPs group results are particularly notable, as they show that the nanosizing of BA and coating with CT dramatically enhanced the gastroprotective effects. This outcome suggests that the encapsulation of Boswellic acid within Chitosan nanoparticles improves their stability, bioavailability, and targeted delivery to the gastrointestinal tract, thereby maximizing their anti-ulcer properties. The use of nanoparticles to deliver Boswellic acids is supported by other studies with the same formulation that have reported enhanced therapeutic efficacy through improved cellular uptake and sustained drug release [45]. In contrast, while showing some improvement compared to the GU group, the BA group did not achieve the same level of protection as the CT/BA NPs group, with preventive indices of 80%. On the other hand, CT/BA NPs significantly enhanced this effect, achieving a preventive index of 96.92% [46]. This highlights the importance of drug formulation strategies in achieving optimal therapeutic outcomes.

### Histological analysis

**Fig. 4:**
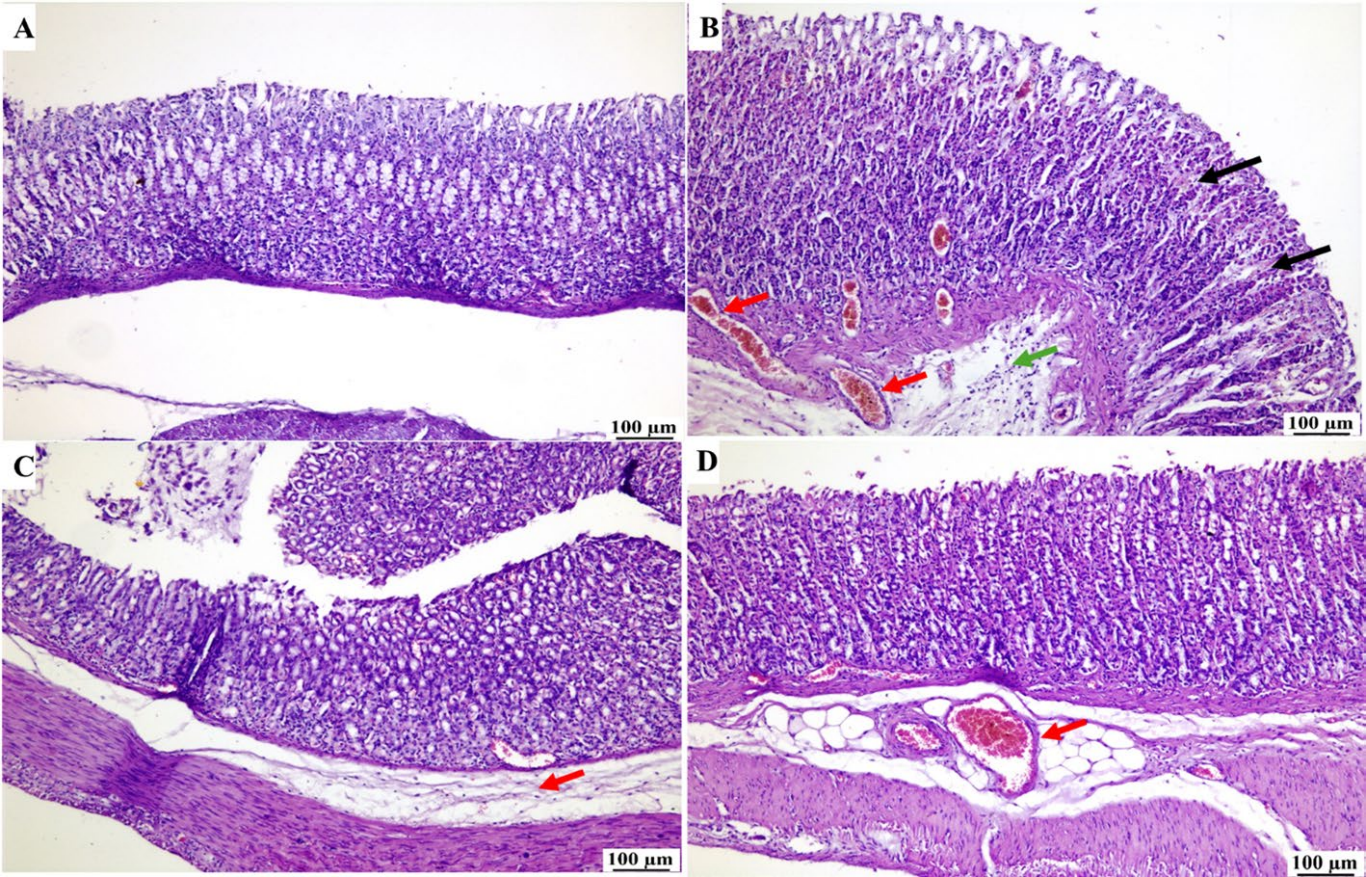
Histopathological examination and microscopic examination of gastric mucosa in **A** Control group showing normal gastric mucosa, **B** GU group showing necrosis in gastric glands (black arrows), numerous congested blood vessels (red arrows) and submucosal inflammatory edema (green arrow), **C** BA-treated group showing exaggerated mucus secretion (black arrow) with mild submucosal edema (red arrow). **D** CT/BA NPs treated group showed apparently normal gastric mucosa with congestion in the submucosa (red arrow)

Microscopic examination of stomach tissues from Control group (C) (Fig. 4A) revealed normal histological structure of the gastric mucosa. The positive control group (GU) (Fig. 4B) showed sloughing of Lamina epithelial necrosis in gastric glands with infiltration of inflammatory cells. Gastric mucosa showed congestion and hemorrhage. The sub-mucosa exhibited inflammatory edema, resulting in the dispersion of sub-mucosal connective tissue. Moderate improvement was noticed in the BA-treated group (Fig. 4C). The examined stomach sections showed increased mucus secretion, mild mononuclear inflammatory cells infiltration in the gastric mucosa, and mild sub-mucosal edema. Apparently, normal gastric mucosa with intact gastric glands was observed in the examined sections from the CT/BA NPs group (Fig. 4D), with congestion or mild submucosal edema in some instances. Similar results were observed in previous work that suggested the protective effects of alpha boswellic acid, yet this work has the novel CT/BA NPs formulation, which shows an enhanced protective effect [10]

### CT/BA NPs modulate different growth factors that promote re-epithelialization and decrease inflammation to stimulate healing

**Fig. 5:**
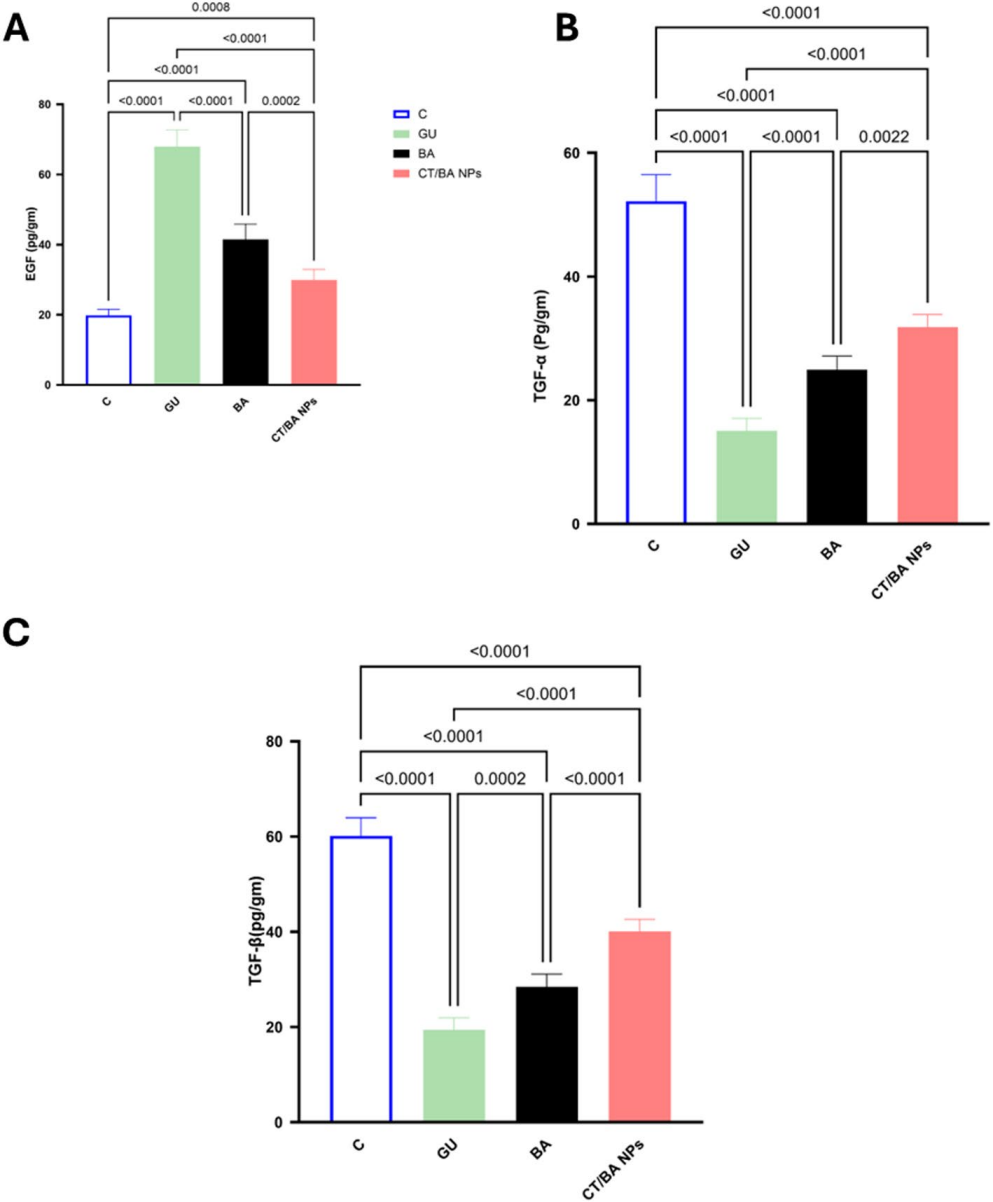
Effect of BA and CT/BA NPs on the levels of growth factors in ethanol-induced gastric ulcer in mice. **A** Epidermal growth factor (EGF), **B** Transforming growth factor alpha (TGF-α). **C** Transforming growth factor beta (TGF-β). Data are expressed as mean ± SD (n = 6). Statistical analysis was performed using one-way ANOVA followed by Tukey’s test

**Fig. 6:**
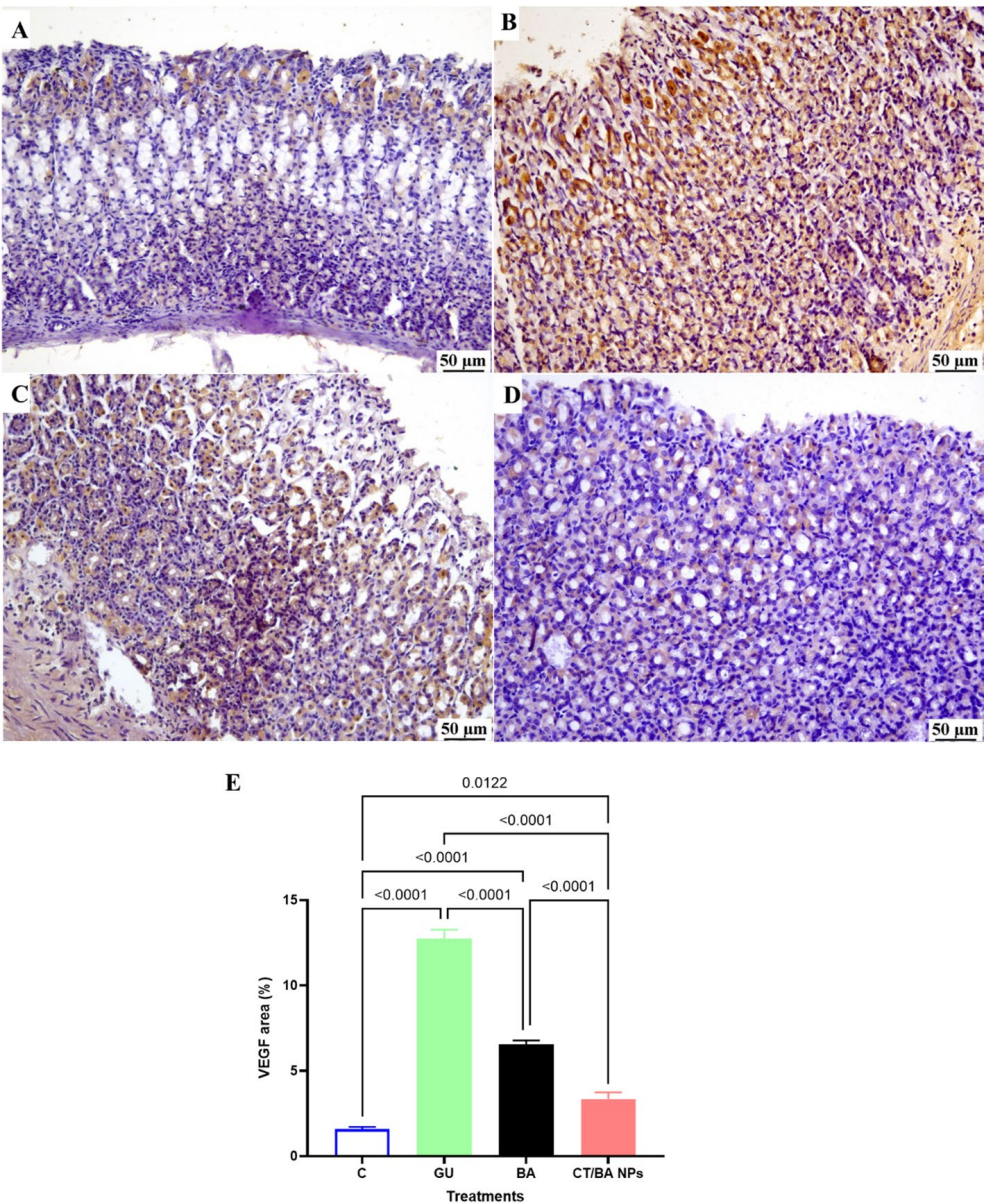
Photomicrograph of immune staining of gastric tissues of **A** normal mice showing weak VEGF expression. **B** GU group showed higher VEGF expression. **C** BA-treated group showing moderate VEGF expression. **D** CT/BA NPs treated group showed moderate VEGF expression. Data are expressed as mean ± SD (n = 6). Statistical analysis was performed using one-way ANOVA followed by Tukey’s test

In this ethanol-induced gastric ulcer model, both BA and CT/BA NPs treatments markedly altered gastric mucosal levels of EGF, TGF-α, and TGF-β1 (Fig. 5A–C) compared to GU; positive control group. Epidermal growth factor (EGF) levels were reduced upon treatment with BA significantly compared to the untreated GU group, whereas the CT/BA NPs-treated group showed a higher decrease by 56% below GU group, a highly significant difference; P = 0.0002. This increase aligns with previous studies showing that when the injury is severe, the body responds by maximally increasing EGF expression to accelerate healing [12, 47]. The decrease in the EGF levels upon exposure to BA suggests that it shows a protective merit and aids in mucosal healing, which halts the need for excessive EGF upregulation [48]. In contrast, the CT/BA NPs formulation resulted in the lowest EGF levels, suggesting the highest protective merit. These findings could be attributed to the mucoadhesive features of the CT/BA NPs that enhanced their residence time and retention in the gastrointestinal tract[49]. TGF-α levels were substantially increased in both treatment groups relative to the GU group. BA increased TGF-α by 66%, and CT/BA NPs caused an even more significant increase with more than a one-fold increase in the levels of TGF-α compared to the unprotected group (*P* < 0.0001). Similarly, TGF-β1 was significantly increased in both groups (approximately 46% increase with BA and more than one-fold increase with CT/BA NP (*P* < 0.0001). The TGF-α was proven to provide dose-dependent protection against stress-induced gastric ulceration [50]. These results indicate that both treatments confer a protective effect on the gastric mucosa by modulating key growth factors, with an edge to the CT/BA NPs due to their Mucoadhesive coatings, allowing for prolonged drug release and increased local drug concentration at the ulcer [51]. This mucosal protective effect mostly occurs through an EGFR-dependent pathway, promoting epithelial proliferation and barrier restoration[6]. Moreover, in the current study, the TGF-β was significantly increased upon treatment with BA and CT/BA NPs by 46.3% and more than one-fold increase, respectively, at (*p* < 0.0001). In fact, TGF-β is a cytokine that is essential for wound healing, promoting fibroblast proliferation, extracellular matrix (ECM) deposition, and tissue remodeling. It promotes healing and has a protective merit in this study [52]. This was in line with a previous study in which TGF-β contributed to gastric ulcer healing along with other growth factors [53]. In addition to that, Vascular endothelial growth factor (VEGF) has a significant role in angiogenesis and gastric ulcer healing [54]. 1.6 fold VEGF immunohistochemical expression differed markedly among the groups (Fig. 6A–E). The normal group showed only weak VEGF positivity, significantly lower than any ulcer-induced group​. In contrast, the untreated ulcer group (GU) exhibited the highest VEGF expression, which is a hallmark of the body's natural response to gastric injury, with intense positive staining that exceeded the normal groups by 7 folds, by which the tissues are trying to compensate for the intense injury to the gastric mucosa as previously described [54]. The BA and CT/BA NPs treatment groups showed moderate VEGF expression—visibly greater than the normal control but notably less than the GU group by 48% and 74%, respectively. The observed patterns of VEGF expression align with those of other growth factors, which demonstrate a superior effect of the CT/BA NPs compared to the normal BA, due to their specific characteristics. Moreover, the decline in the VEGF expression in the gastric mucosa suggests a protective effect of the BA against ethanol-induced gastric ulcer, with an edge of the newly formulated CT/BA NPs over the normal BA. This can be attributed to VEGF's essential role as a cellular defense against injury by enhancing angiogenesis, promoting epithelial cell survival, and restoring microcirculation [55].

### CT/BA NPs exert their protective effect through RAS-ERK signaling as well as modulation of proinflammatory cytokines

**Fig. 7:**
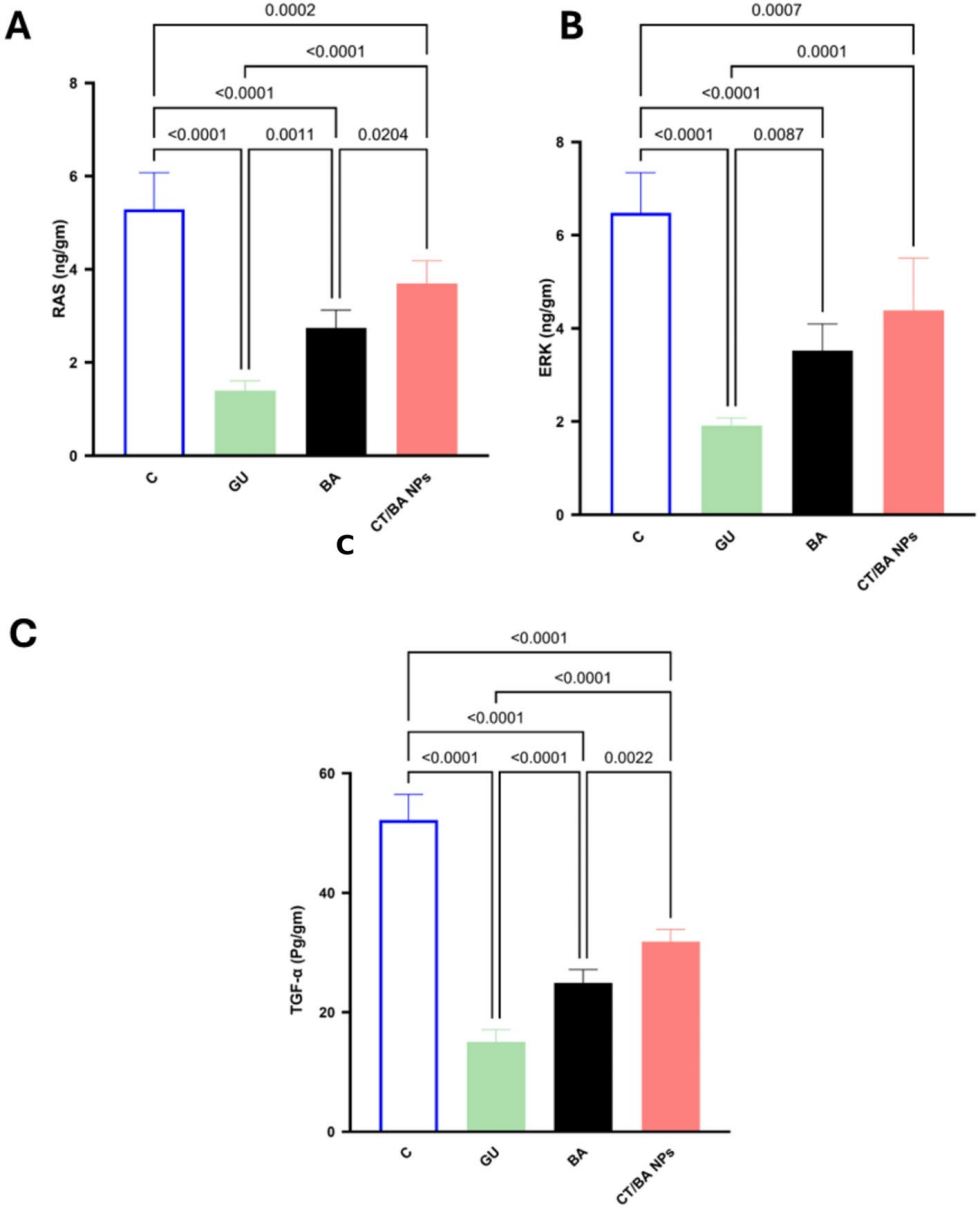
Effect of BA and CT/BA NPs on the levels of RAS/ERK. **A** Rat sarcoma viral oncogene homolog (RAS) **B** Extracellular signal-regulated kinase (ERK) **C** Tumor necrosis factor alpha (TNF-α). Data are expressed as mean ± SD (n = 6). Statistical analysis was performed using one-way ANOVA followed by Tukey’s test

In the current study, the preventive treatment in both groups showed higher RAS-ERK signaling, which aligns with previous research that showed that healing tissue at the ulcer margin exhibits heightened RAS-ERK signaling, reflecting the activation of this pathway as a natural repair mechanism [56] As presented in Fig. 7A–C,7 The levels of RAS were significantly decreased in the groups treated with the BA and the group that administered CT/BA NPs as protective medication by 95% and 1.6 folds; respectively at p < 0.0001, compared to the positive control group. By the same token, ERK showed a significant decline upon treatment with both BA and CT/BA NPs by 83% and 1.2-fold, respectively, compared to the GU-treated group at *p* < 0.0001. This aligns with previous work suggesting that the RAS-ERK pathway is involved in gastric homeostasis [57]. Moreover, the activity of the RAS-ERK pathway is orchestrated via the EGF. This indicates that the pathway is responsive to growth factors, which are known to promote tissue repair and regeneration [58]. In fact, augmenting the RAS-ERK pathway is reflected in the possible involvement of BA and its new formula as a protective agent against gastric ulcers in highly susceptible individuals due to its role in promoting cell proliferation and tissue repair [59].

Finally, the CT/BA NPs formula was found to decrease significantly the proinflammatory cytokine TNF-α in a manner that exceeds the BA itself at *P* < 0.0001. These findings strongly support the efficacy of our formula as a promising protective agent against gastric ulcers, as 76.476.4

TNF-α contributes to ulcer formation by promoting leukocyte recruitment and increasing the expression of chemokines. It also elevates oxidative stress markers and inflammatory cytokines, exacerbating gastric mucosal damage [51, 52].

The observed downregulation of RAS and ERK in BA and CT/BA NP groups aligns well with earlier work reporting the importance of this pathway in gastric mucosal repair. A previous study emphasized that ulcer healing is critically dependent on growth factor signaling through EGFR, which in turn activates Ras–ERK cascades to stimulate epithelial proliferation and tissue regeneration [12]. Similarly, another study highlighted the central role of ERK1/2 in regulating cell survival and apoptosis, which are two processes essential for maintaining gastric homeostasis [13]. Furthermore, a different study showed that Raf-1 activation during experimental ulcer healing is Ras-mediated, highlighting the mechanistic link between Ras–ERK signaling and mucosal compensation [49]. Together, these findings reinforce our results, suggesting that modulation of Ras–ERK signaling by CT/BA NPs helps to balance proliferation and inflammation, thereby accelerating mucosal recovery in ethanol-induced ulcers.

### Toxicity assessment

**Fig. 8:**
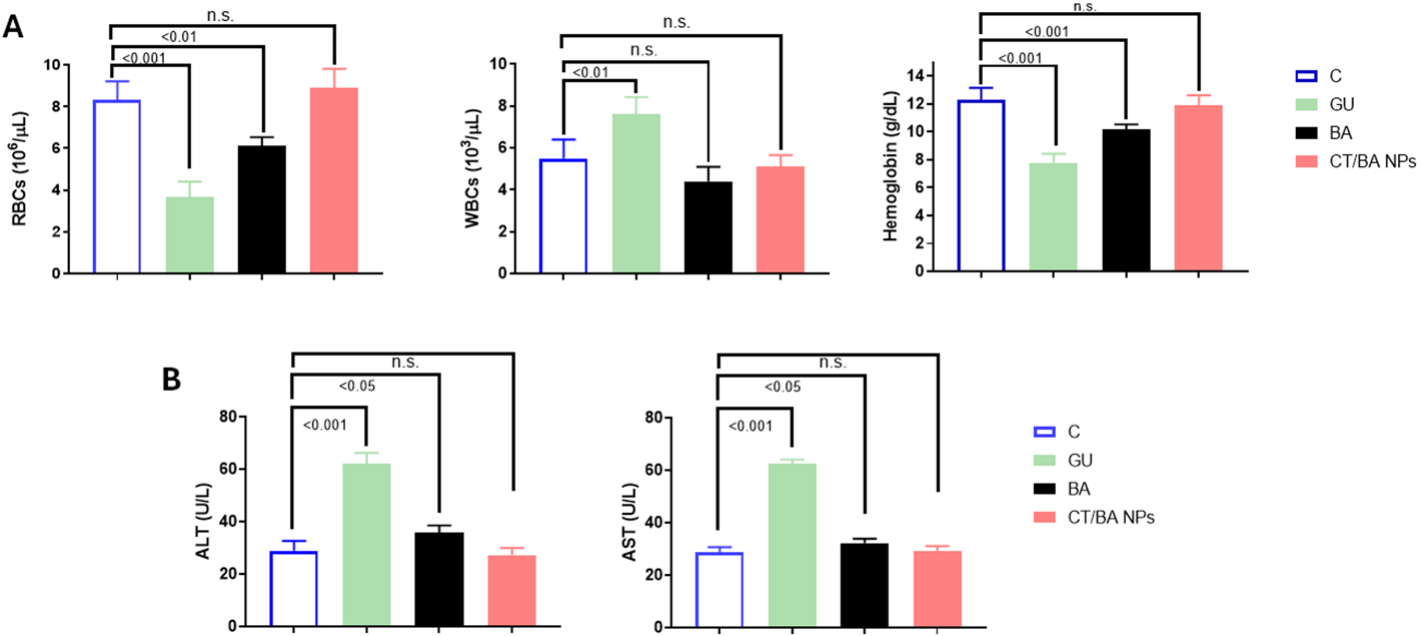
**A** Hematological parameters evaluation, showing the RBCs, WBCs, and hemoglobin levels in blood samples collected from control, ethanol-induced gastric ulcer mice, BA, and CT/BA NPs treated mice on day 24. **B** Serum levels of the liver enzymes ALT and AST in blood samples collected from the mice on day 24. The results are the mean value ± S.D. (n = 6)

The hematological parameters were evaluated on day 24 after GU induction (Fig. 8A). The average Hb level in normal mice was 12.3 ± 0.85 g/dL. Mice with GU not treated with either BA or CT/BA NPs experienced a significant drop in Hb levels, measuring 7.18 ± 0.59 g/dL. BA-treated mice exhibited higher Hb levels, reaching 10.2 ± 0.31 g/dL. The CT/BA NPs treatment led to a remarkable increase in Hb levels to 11.9 ± 0.73, approaching normal levels. The reduction in Hb levels in untreated GU mice suggested noteworthy anemia due to gastrointestinal bleeding. CT/BA NPs treatment exhibited a notable enhancement in Hb levels, implying its potential to relieve the hematological impact of GI bleeding [38, 60].

Additionally, the GU mice that did not receive treatment exhibited a significant decrease in RBCs of 3.65 ± 0.0.75 × 10^6^/μL, due to GI bleeding, contrary to the control mice with a red blood cell count of 8.3 ± 0.9 × 10^6^/μL. The BA remarkably raised the RBC to 6.1 ± 0.4 × 10^6^/μL. However, the CT/BA NPs treatment fixed the RBC count to close-normal levels at 8.9 ± 0.9 × 10^6^/μL.

Mice treated with CT/BA-NPs exhibited a WBC count of 5.1 ± 0.6 × 10^3^/μL, almost close to the levels detected in control mice. On the other hand, untreated GU mice showed an apparent increase in WBCs owing to the inflammatory response to mucosal damage [61]. The near-normal Hb levels, RBCs, and WBCs counts demonstrate the safety and curative effects of BA and CT/BA NPs.

Moreover, untreated GU mice had remarkably higher AST levels, indicating substantial hepatic stress caused by ethanol exposure [62]. Nonetheless, the mice group treated with BA and CT/BA NPs demonstrated lower ALT and AST levels than those observed in untreated GU mice, indicating reduced hepatic stress.

## Conclusion and future directions

In conclusion, this study demonstrates that chitosan-coated Boswellic acids anoparticles (CT/BA NPs) exhibit superior gastroprotective effects compared to uncoated BA, primarily due to their enhanced mucoadhesion and retention within the gastric mucosa. Histological analysis revealed reduced inflammation, edema, and necrosis in treated groups, with CT/BA NPs preserving the most intact gastric architecture. Furthermore, growth factor modulation showed significant increases in TGF-α and TGF-β1, alongside controlled VEGF expression, collectively promoting mucosal regeneration. The RAS-ERK signaling pathway was markedly downregulated in treated groups, further supporting its role in maintaining gastric homeostasis and facilitating tissue repair. These findings highlight CT/BA NPs as a promising therapeutic platform for enhancing gastric mucosal protection and preventing ethanol-induced ulceration. Looking ahead, future studies should extend these investigations to include diverse ulcer models to establish the generalizability of the findings. Additionally, early-phase clinical trials are warranted to evaluate the safety, tolerability, and preliminary efficacy of CT/BA NPs in human subjects, bridging the gap between preclinical research and clinical application. Successful translation of nanoparticle-based therapies from bench to bedside will also require addressing critical challenges in formulation scalability. Efforts should focus on ensuring consistent particle size, encapsulation efficiency, and physicochemical stability during large-scale production. Finally, optimizing manufacturing protocols, such as refining solvent evaporation techniques and mixing dynamics, will be crucial for achieving batch-to-batch reproducibility and regulatory compliance.

## Supplementary Information

Below is the link to the electronic supplementary material.

Supplementary Material 1

## Data Availability

All data generated or analysed during this study are included in this published article [and its supplementary information files.
