# Supplementary material for: Mucoadhesive chitosan-coated boswellic acids nanoparticles as promising gastroprotective nanoagents via modulation of the RAS/ERK signaling pathway
Source: Discov Nano. 2025 Oct 23;20(1):190. doi: 10.1186/s11671-025-04375-8 (PMC12549511; doi:10.1186/s11671-025-04375-8)
Supplement: Supplementary file 1 — Supplementary Material 1 [file 11671_2025_4375_MOESM1_ESM.docx]

**Supplementary Materials**

*Extraction and chemical characterization of the major chemical components of Boswellia sacra resin were conducted as per our previously published paper [1].*

The major chemical components of *Boswellia sacra* oleo-gum resin (BSR) were extracted using methanol to obtain BME following our previously reported method [1]. BSR pieces were ground, and 100 mg of the ground material was added to 300 mL of methanol. Then, the mixture was stirred at room temperature for 24 hours in a Schott bottle. The mixture was filtered twice, and the methanol was evaporated under vacuum using a rotary evaporator. The resulting BME powder was purified by redissolving in methanol, and the solution was filtered through a syringe filter (pore size: 0.22 µm). The clear solution was then placed in an oven at 60°C until the BME powder was completely dry and the solvent was evaporated. The primary chemical constituents of BME were identified using liquid chromatography-electrospray ionization-tandem mass spectrometry (LC/ESI-MS/MS) with an X500R LC-QTOF mass spectrometer (SCIEX, USA). Separation was performed on an Inertsil C18 column (25 cm × 4.6 mm × 5 µm). The mobile phases consisted of A: (80:20) methanol and B: 0.1% formic acid. The gradient elution was programmed as follows: 3% B from 0 to 5 minutes, 3-90% B from 5 to 18 minutes, 90% B from 18 to 23 minutes, 90-3% B from 23 to 27 minutes, and 3-1% B from 27 to 30 minutes. The sample volume was 6 µL, and the flow rate was set at 1.0 mL/min. Negative ionization mode was used for MS/MS analysis, with SWATH scanning from 50 to 1000 Da and the following parameters: curtain gas at 30 psi, IonSpray voltage at 5000 V, source temperature at 500°C, ion source gases 1 and 2 at 50 psi each, declustering potential at 80 V, and collision energy at 10 V. Compound identification was performed by comparing results to the NIST Library, a built-in reference library.


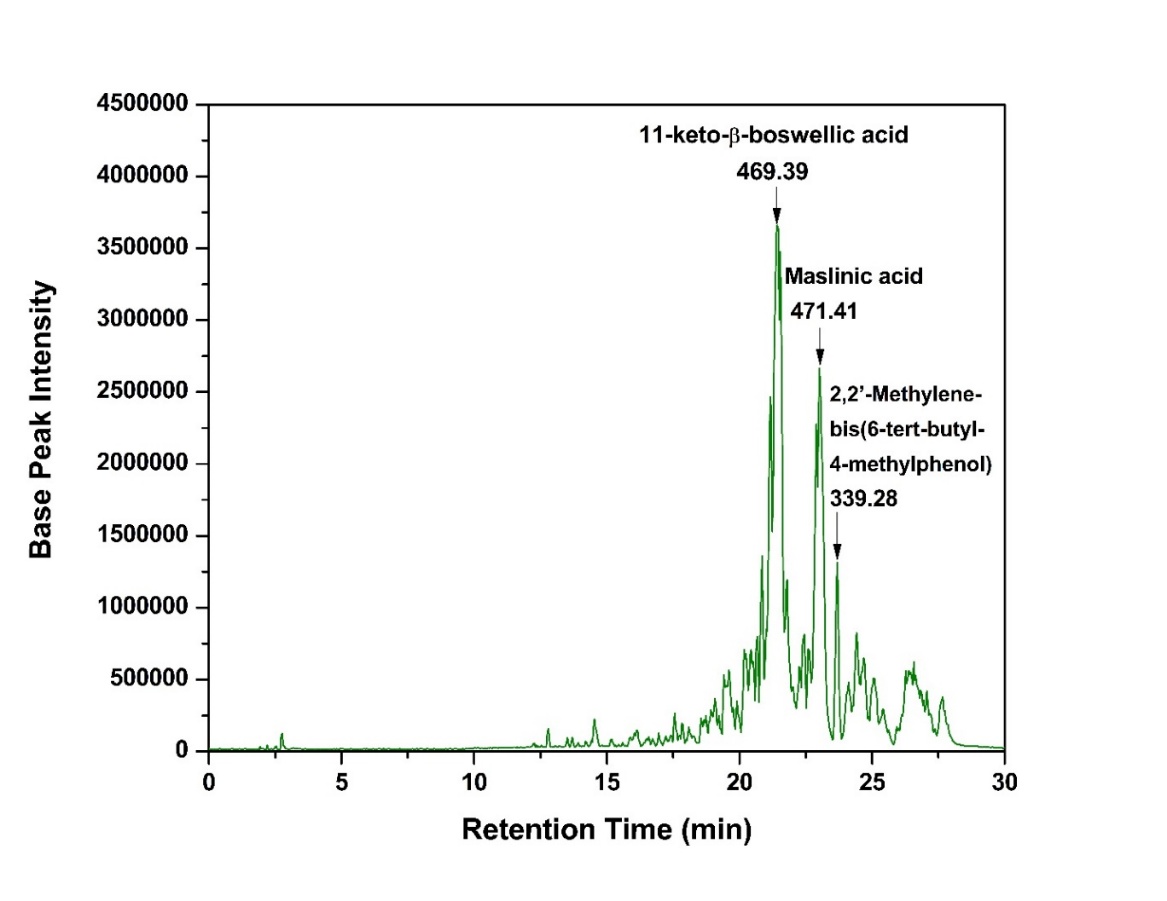


**Figure S1.** LC-ESI-MS/MS ion chromatogram (in the negative ion mode) of the *Boswellia sacra* oleo-gum resin methanolic extract, as previously reported in our previous study[1].

**Table S1.** Identified Peaks in the LC-MS Spectrum of the BME, reported in our previous study [1].

| **Peak** | **Rt** | **Assignment** | **Precursor ion (m/z)** | **Molecular Formula** | **Productions MS/MS** | **Class** |
| --- | --- | --- | --- | --- | --- | --- |
|  | 2.65 | Quinic acid | 191.0455 | C_7_H_12_O_6_ | 191, 173, 147, 127, 109, 93, 87, 85, 67 | Organic acid |
|  | 2.77 | D-(+)-Trehalose | 341.1519 | C_12_H_22_O_11_ | 341, 211, 179, 89 | Disaccharide |
|  | 2.84 | Quinic acid | 191.0819 | C_7_H_12_O_6_ | 191, 173, 147, 127, 109, 93, 87, 85, 67 | Organic acid |
|  | 3.11 | Ureidosuccinic acid | 174.9804 | C_5_H_8_N_2_O_5_ | 175, 132 | Organic acid |
|  | 3.18 | Quinic acid | 191.0457 | C_7_H_12_O_6_ | 191, 173, 147, 127, 109, 93, 87, 85, 67 | Organic acid |
|  | 5.64 | Methylmalonic acid | 117.0362 | C_4_H_6_O_4_ | 117, 116, 115 | Organic acid |
|  | 10.56 | Oxalacetic acid | 131.0534 | C_4_H_4_O_5_ | 131, 130, 129, 128 | Organic acid |
|  | 10.64 | Neochlorogenic acid | 353.1439 | C_16_H_18_O_9_ | 353, 259, 191, 180 135, 134 | Phenolic acid |
|  | 10.79 | Tridecanoyl-sn-glycero-3-phosphate | 367.2089 | C_21_H_44_NO_7_P | 367, 311, 215, 153, 123 | Phospholipid |
|  | 10.83 | Neochlorogenic acid | 353.0947 | C_16_H_18_O_9_ | 353, 259, 191, 180 135, 134 | Phenolic acid |
|  | 11.05 | Neochlorogenic acid | 353.1336 | C_16_H_18_O_9_ | 353, 259, 191, 180 135, 134 | Phenolic acid |
|  | 11.24 | 2-(2-Hydroxyethoxy)phenol | 153.0406 | C_8_H_10_O_3_ | 153, 150, 109, 91 | Phenol |
|  | 12.80 | Gabapentin-related compound E | 185.1069 | C_9_H_14_O_4_ | 185, 142, 141, 123, 99, 81, 71, 57 | Organic acid |
|  | 14.54 | 6-Fluoro-4-hydroxycoumarin | 381.1810 | C_9_H_5_FO_3_ | 381, 309, 180, 179, 136, 135, 94 | Hydroxycoumarin |
|  | 15.07 | 2’-Hydroxy-4’-methoxyacetophenone | 165.0776 | C_9_H_10_O_3_ | 165, 150, 122, 108, 71 | Phenol |
|  | 15.22 | Madecassic acid | 503.4005 | C_30_H_48_O_6_ | 503, 499, 443, 371, 248 | Triterpene |
|  | 15.71 | 2’-Hydroxy-4’-methoxyacetophenone | 165.0785 | C_9_H_10_O_3_ | 165, 150, 122, 108, 71 | Phenol |
|  | 16.01 | Madecassic acid | 503.3997 | C_30_H_48_O_6_ | 503, 443, 399 | Triterpene |
|  | 16.35 | Maslinic acid | 471.3008 | C_30_H_48_O_4_ | 471, 427, 397, 353, 314, 263, 217, 189, 145, 113 | Triterpene |
|  | 16.47 | 4-Androsten-17.beta-ol-3-one sulfate | 367.2594 | C_19_H_27_O_5_S | 367, 287, 243, 85 | Steroid |
|  | 17.15 | 7,7-Dimethyl-(5Z,8Z)-eicosadienoic acid | 335.2658 | C_22_H_40_O_2_ | 335, 291 | Fatty acid |
|  | 17.26 | N-2-Hydroxyethylpiperazine | 251.1983 | C_6_H_14_N_2_O | 251 | Alkaloid |
|  | 17.34 | 3-Phenylbutyric acid | 163.0985 | C_10_H_12_O_2_ | 163, 148, 134 | Organic acid |
|  | 17.34 | Thomboxane B3 | 367.2588 | C_20_H_32_O_6_ | 367, 352, 331, 251, 230, 170, 169, 122, 97 | Eicosanoid |
|  | 17.60 | (+)-trans-Chrysanthemic acid | 167.1306 | C_10_H_16_O_2_ | 167, 133, 109 | Monoterpene |
|  | 17.87 | cis-4,10,13,16-Docosatetraenoic acid | 331.2335 | C_22_H_36_O_2_ | 331, 288, 287, 236, 83 | Fatty acid |
|  | 17.98 | Genkwanin | 283.0990 | C_16_H_12_O_5_ | 283, 268, 251, 179, 135, 79 | Flavonoid |
|  | 22.33 | 4-chloro-alpha-(4-chlorophenyl)-Benzeneacetic acid | 279.2334 | C_14_H_9_Cl_2_O_2_^-^ | 279, 236, 235, 199, 183, 153, 134, 97, 71 | Organic acid |
|  | 22.90 | 11-Keto-beta-boswellic acid | 469.3891 | C_30_H_46_O_4_ | 469, 452, 407, 391, 376, 271, 61 | Triterpene |
|  | 23.58 | Trihydroxycholestanoic acid | 449.3703 | C_27_H_46_O | 449, 327 | Bile acid |
|  | 23.70 | 2,2’-Methylene-bis(6-tert-butyl-4-methylphenol) | 339.2758 | C_23_H_32_O_2_ | 339, 327, 165, 164, 163, 147 | Phenol |
|  | 24.38 | 3beta,7alpha-Dihydroxy-5-cholestenoic acid | 431.3579 | C_27_H_44_O_4_ | 431 | Bile acid |
|  | 24.53 | Maslinic acid | 471.4052 | C_30_H_48_O_4_ | 471 | Triterpene |
|  | 26.57 | 3-Acetyl-11-keto-beta-boswellic acid | 511.4039 | C_32_H_48_O_5_ | 511, 60, 59 | Triterpene |
|  | 27.37 | 3-Acetyl-11-keto-beta-boswellic acid | 511.4054 | C_32_H_48_O_5_ | 511, 60, 59 | Triterpene |

**References**

1. S. A. Fahmy *et al.*, “Synergistic Enhancement of Carboplatin Efficacy through pH-Sensitive Nanoparticles Formulated Using Naturally Derived Boswellia Extract for Colorectal Cancer Therapy,” *Pharmaceutics*, vol. 16, no. 10, p. 1282, Oct. 2024, doi: 10.3390/PHARMACEUTICS16101282/S1.
